# The Elephant in the Room: Clinical Progression and Management of Elephant Ear Plant Toxicity in an Adult

**DOI:** 10.7759/cureus.66445

**Published:** 2024-08-08

**Authors:** Jordan C Malone, Anirudha Chatterjee, Keegan Colletier, Marc Shabot

**Affiliations:** 1 Internal Medicine, University of Texas Medical Branch at Galveston, Galveston, USA; 2 Gastroenterology and Hepatology, University of Texas Medical Branch at Galveston, Galveston, USA

**Keywords:** calcium oxalate crystals, elephant ear, esophagogastroduodenoscopy (egd), acute dysphagia, plant ingestion

## Abstract

Elephant ear plants are popular ornamental plants renowned for their large foliage. These plants have been implicated in various inadvertent and deliberate ingestions. The leaves and roots of these plants contain raphides, which are needle-shaped calcium oxalate crystals. Ingestion of these crystals results in a localized inflammatory response, typically manifesting as irritation, edema, hypersalivation, and dysphagia. Herein, we describe a case of an older gentleman who presented to our institution following intentional ingestion of the leaves and roots of an elephant ear plant. This report describes the clinical manifestations secondary to the toxicities related to the ingestion of this plant and displays the successful conservative management approach employed following multiple diagnostic studies.

## Introduction

The genera *Colocasia*, *Alocasia*, and *Xanthosoma*, collectively known as “Elephant Ears,” are popular ornamental plants originating from southern Asia, the Pacific islands, and tropical America that are now grown worldwide and are renowned for their large foliage. These plants have been implicated in various inadvertent ingestions, primarily involving pediatric patients [1-3]. The leaves and roots of these plants contain raphides, which are needle-shaped calcium oxalate crystals. Ingestion of these crystals without first cooking the plant results in a localized inflammatory response, typically manifesting as irritation, edema, hypersalivation, and dysphagia [2,4]. Herein, we describe a case of a 60-year-old man who presented with symptoms of drooling, oropharyngeal pain, and dysphagia following deliberate ingestion of the leaves and roots of an elephant ear plant.

## Case presentation

A 60-year-old male with a medical history of hypertension presented to the emergency department of our large academic center in southern Texas in January of 2024 reporting an allergic reaction following the ingestion of the leaves and root of the elephant ear plant shortly before his presentation. He experienced a burning sensation on the tongue, oral pain, and difficulty and pain with swallowing. Initial vital signs revealed significant hypertension (193/123 mmHg) and tachycardia (heart rate of 119 beats per minute). Physical examination identified an anxious, actively drooling patient. An oropharyngeal exam showed mild erythema without laryngeal edema. Pulmonary examination was unremarkable with clear lung sounds, normal work of breathing, and oxygen saturations maintained above 95% on room air. Laboratory investigations were unremarkable. He was given intramuscular epinephrine, intravenous methylprednisolone, and famotidine, along with an oral swish-and-swallow solution containing viscous lidocaine, diphenhydramine, and an antacid mixture (aluminum hydroxide, magnesium hydroxide, and simethicone). Poison control was contacted and relayed that treatment should focus on supportive, symptom-targeted care. Once he was able to tolerate oral intake he could be safely discharged home with outpatient follow-up as admission for observation was only warranted should his symptoms worsen or not improve. He was observed for four hours following administration of the above medications. His symptoms gradually improved to the point where he could tolerate oral intake. He subsequently felt comfortable being discharged home. Esophagogastroduodenoscopy (EGD) was deferred to the outpatient setting given his improvement in symptoms. An expedited outpatient follow-up with gastroenterology was arranged on discharge.

**Figure 1:**
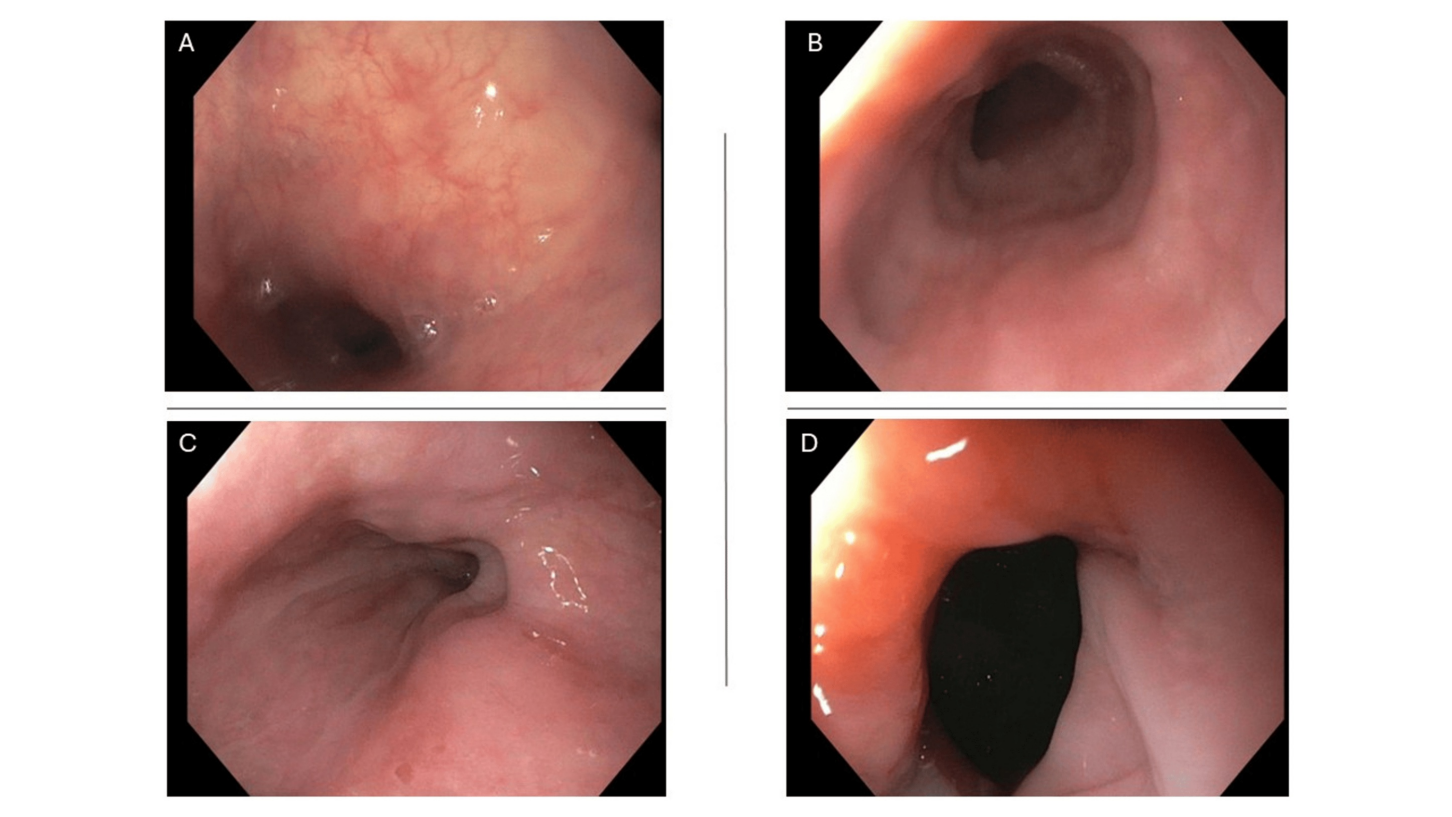
Esophagogastroduodenoscopy images without grossly identified pathological change. A: Upper third of the esophagus. B: Middle third of the esophagus. C: Lower third of the esophagus. D: Gastroesophageal junction.

At his gastroenterology clinic follow-up one and a half weeks post discharge, he reported persistent but intermittent “squeezing” episodes of lower chest and upper abdominal pain since his emergency department visit. Although his oral pain and odynophagia had resolved, intermittent dysphagia persisted. EGD and esophageal manometry were scheduled. He was prescribed pantoprazole twice daily, sucralfate, and viscous lidocaine while awaiting upper endoscopy. Subsequent esophageal manometry revealed non-specific segmental esophageal spasms. EGD the same day showed an unremarkable esophagus (Figure 1). Random gastric biopsies were taken from the gastric body, incisura, and antrum for *Helicobacter pylori *testing. No other gross endoscopic abnormalities were identified within the upper gastrointestinal tract.

At a follow-up visit two weeks later, he reported a decrease in the frequency of his chest pressure episodes and a return to baseline health. His random gastric biopsies taken during EGD returned positive for *H. pylori* organisms for which he was started on quadruple therapy (bismuth, pantoprazole, metronidazole, and tetracycline) based on local resistance patterns.

## Discussion

The elephant ear plant is traditionally purported to have numerous health benefits, including antimicrobial, anti-inflammatory, and cytotoxic properties, though evidence supporting these claims is limited. The plant is considered edible only when thoroughly cooked or boiled to degrade the raphide crystals [5]. Ingestion of raw elephant ear plants and the resultant toxicities are predominantly accidental and documented largely in pediatric populations. This case illustrates the dose-dependent toxic effects of raw ingestion in an adult, attributed mainly to localized irritation and inflammation from direct exposure to calcium oxalate crystals [4,6]. Our patient’s symptoms were managed effectively with supportive care, but this case highlights the importance of serial respiratory assessments, as upper airway obstruction necessitating endotracheal intubation has been reported [6]. Our patient’s upper endoscopy and esophageal manometry did not reveal significant findings related to his toxic ingestion. We believe that the *H. pylori* organisms identified and treated were incidental and unrelated to our patient's initial presentation as evidenced by his clinical course. This case underscores the clinical value of a conservative, symptomatic management approach for this plant toxicity in adults.

## Conclusions

Though most published reports describing ingestion of the elephant ear plant involve pediatric patients, it is imperative to contribute rarer cases of such ingestion in adults. The most worrisome complication following ingestion of this plant is airway compromise necessitating endotracheal intubation and mechanical ventilation, stressing the importance of immediate and serial airway patency examinations. Clinicians who treat adults should be aware of the potential toxicities related to ingestion of this plant, along with the typical clinical progression. Diagnostic studies were completed in this case but were found to be non-specific. Modalities such as EGD and esophageal manometry can be considered to rule out other organic causes. However, clinical history should be heavily weighed in the setting of reported deliberate ingestion of such a plant. It is essential to evaluate for syndromes with overlapping symptoms that may require more emergent intervention, especially anaphylactoid reactions. This case serves as an example of how this overlap may present in real practice as our patient was initially treated for such. However, we believe the antacid and local anesthetic cocktail that was administered likely contributed the most to his symptomatic improvement as opposed to the empiric anaphylaxis treatment. This report illustrates a case of cellular mechanical injury caused by a direct physical irritant, resulting in a localized inflammatory reaction rather than a true hypersensitivity reaction, similar to the mechanism observed in irritant contact dermatitis. The clinical focus should continue to target symptomatic management and further avoidance of the irritant, which, in this case, is the uncooked roots and leaves of the elephant ear plant.
